# Beyond Event-Free Survival: A Multi-State Analysis of Local Recurrence, Metastasis, and Death in Soft Tissue Sarcoma

**DOI:** 10.3390/cancers18213414

**Published:** 2026-10-22

**Authors:** Markus Schärer, Philip Heesen, Gabriela Studer, Bettina Vogel, Bruno Fuchs, on behalf of the Swiss Sarcoma Network

**Affiliations:** 1 Sarcoma Service, Department of Orthopedics and Trauma, Kantonsspital Winterthur, 8400 Winterthur, Switzerland; 2 Department of Hip and Knee Surgery, Schulthess Clinic Zurich, 8008 Zurich, Switzerland; 3 Department of Neuroradiology, Heidelberg University, D-69117 Heidelberg, Germany; 4 Department of Biostatistics at the Epidemiology, Biostatistics and Prevention Institute, University of Zurich, 8001 Zurich, Switzerland; 5 Faculty of Health Sciences & Medicine, University of Lucerne, Frohburgstrasse 3, 6002 Luzern, Switzerland; 6 Sarcoma Service, Department of Radiation Oncology, Sarcoma Center, LUKS University Hospital, 6000 Lucerne, Switzerland; 7 Sarcoma Service, Department of Orthopedics and Trauma, Sarcoma Center, LUKS University Hospital, 6000 Lucerne, Switzerland

**Keywords:** soft tissue sarcoma, local recurrence, distant metastasis, event-free survival, multi-state model, disease progression, time-dependent analysis

## Abstract

Background/Objectives: Event-free survival (EFS) is widely used in soft tissue sarcoma (STS) research but combines clinically distinct outcomes such as local recurrence (LR), distant metastasis (DM), and death into a single endpoint. We aimed to decompose conventional EFS and characterize STS progression as a longitudinal process of transitions between clinically distinct disease states. Methods: Prospectively and retrospectively collected registry data from 662 patients with STS at two tertiary sarcoma centers were retrospectively analyzed. EFS was decomposed by first-event type, and longitudinal disease evolution was assessed using multi-state modeling and time-dependent Cox regression. Results: Among 585 patients included in the EFS analysis, first events were isolated LR in 114 (19.5%), DM in 106 (18.1%), synchronous LR and DM in 21 (3.6%), and death without documented recurrence in 19 (3.2%). In the full cohort, 151 deaths occurred. After multivariable adjustment, isolated LR showed a moderate but statistically imprecise association with mortality compared with the no documented LR or DM state (HR 2.55, 95% CI 0.93–7.02), whereas substantially greater mortality hazards were observed for isolated DM (HR 17.56, 95% CI 9.46–32.60) and combined LR and DM (HR 32.63, 95% CI 16.86–63.17). Conclusions: STS progression is characterized by clinically distinct, evolving disease states with markedly different prognostic implications. A disease-state framework complements conventional EFS by preserving the nature and longitudinal evolution of recurrence and may provide a more informative representation of STS progression.

## Simple Summary

Soft tissue sarcomas are rare cancers that can develop local recurrence, metastasis, or both after treatment. These different events are often combined into a single outcome in clinical studies, although they may have very different consequences for patients. In this study of 662 patients, we examined how patients moved between different disease states over time and how mortality risk differed according to their current disease state. Local recurrence alone had a comparatively limited association with mortality, whereas metastatic disease was associated with a substantially higher risk of death, particularly when local recurrence and metastasis coexisted. Our findings show that soft tissue sarcoma can be viewed as an evolving disease process rather than solely by the occurrence of a first recurrence. This approach may help future research provide more informative prognostic assessment and better reflect how patients’ disease evolves over time.

## 1. Introduction

Soft tissue sarcomas (STS) comprise a heterogeneous group of rare mesenchymal malignancies characterized by diverse biological behavior and clinical outcomes [1,2]. Despite advances in surgery, radiotherapy, and systemic therapy, recurrence remains the principal determinant of long-term survival [3,4,5]. Following treatment of localized disease, patients may remain recurrence-free, develop local recurrence (LR), distant metastasis (DM), or both. Consequently, recurrence-related endpoints such as disease-free survival (DFS) and event-free survival (EFS) have become central outcome measures in both clinical trials and observational sarcoma research [6,7,8,9,10].

Despite their widespread use, conventional DFS and EFS summarize disease progression as a single composite endpoint. LR, DM, synchronous locoregional and systemic progression, and death without documented recurrence are all considered equivalent events despite representing distinct biological processes with different therapeutic and prognostic implications [11,12]. Although composite endpoints improve statistical efficiency, they may obscure clinically meaningful heterogeneity in disease evolution by collapsing fundamentally different patterns of progression into a single outcome measure [13]. From a clinical perspective, an isolated LR that remains amenable to salvage treatment differs fundamentally from the development of systemic metastatic disease, yet both contribute equally to conventional recurrence-free survival metrics [9,14,15,16,17].

Previous studies have demonstrated that recurrence characteristics influence prognosis beyond the mere occurrence of recurrence. Early local recurrence is consistently associated with inferior survival compared with late local recurrence, while the timing of recurrence itself has emerged as an independent prognostic factor [5,17,18,19,20,21]. Likewise, multi-state analyses have shown strong associations between LR, metastatic progression, and death, highlighting the dynamic relationship between successive disease events [22,23]. Nevertheless, most studies continue to evaluate recurrence as a binary outcome or focus on individual recurrence events in isolation, providing only limited insight into how patients transition between clinically distinct disease states over time [24].

More recently, we reported a phenotype-based analysis of the same prospective Swiss Sarcoma Network (SSN) cohort, in which patients were classified according to distinct recurrence phenotypes defined by the presence, timing, and sequence of LR and DM [25]. That study established clinically distinct recurrence phenotypes and characterized their clinicopathological features and survival outcomes. However, it did not examine how individual patients transitioned between disease states over time, decompose conventional EFS into its individual first-event components, or evaluate mortality according to patients’ current disease state. These unresolved questions provide the rationale for the present complementary longitudinal analysis.

Consequently, an important conceptual gap remained in the current understanding of STS progression. While our previous work established clinically distinct recurrence phenotypes, it did not investigate the dynamic process through which patients transition between disease states over time. Patients may follow markedly different clinical courses, including isolated LR, isolated DM, sequential LR followed by DM, DM followed by LR, synchronous recurrence, or metastatic disease at presentation [4,26]. These patterns represent distinct pathways of disease evolution with potentially different biological behavior and prognostic implications [5,9]. However, conventional DFS and EFS frameworks reduce these heterogeneous disease courses to a single composite endpoint, limiting their ability to capture the longitudinal nature of disease progression and its evolving prognostic significance [27].

We therefore aimed to characterize STS progression as a sequence of dynamic disease states by decomposing conventional EFS into its component first-event types and reconstructing longitudinal disease trajectories using disease-state classification, multi-state modeling, and time-dependent survival analyses. We hypothesized that the prognostic significance of recurrence differs according to event type and the patient’s evolving disease state. By viewing recurrence as a dynamic process rather than a single endpoint, we sought to establish a more comprehensive framework for understanding disease evolution and prognostic assessment in STS.

## 2. Materials and Methods

### 2.1. Study Design and Data Source

This retrospective multicenter cohort study used data from two tertiary sarcoma centers participating in the Swiss Sarcoma Network. The dataset comprised both prospectively recorded clinical information and retrospectively abstracted medical-record data. Consecutive adult patients with STS treated between January 2015 and December 2025 were screened for inclusion. Clinical, pathological, treatment-related, and follow-up data were extracted from the institutional registries and verified through electronic medical record review when necessary. The source cohort has previously been reported in a phenotype-based analysis of the SSN registry [25]. The present study represents a complementary longitudinal analysis focusing on EFS decomposition, disease-state transitions, and time-dependent mortality.

### 2.2. Study Population

Patients with histologically confirmed STS who underwent treatment at one of the two participating SSN centers during the study period were eligible for inclusion. Patients were identified from the SSN registry and screened according to predefined eligibility criteria.

**Figure 1:**
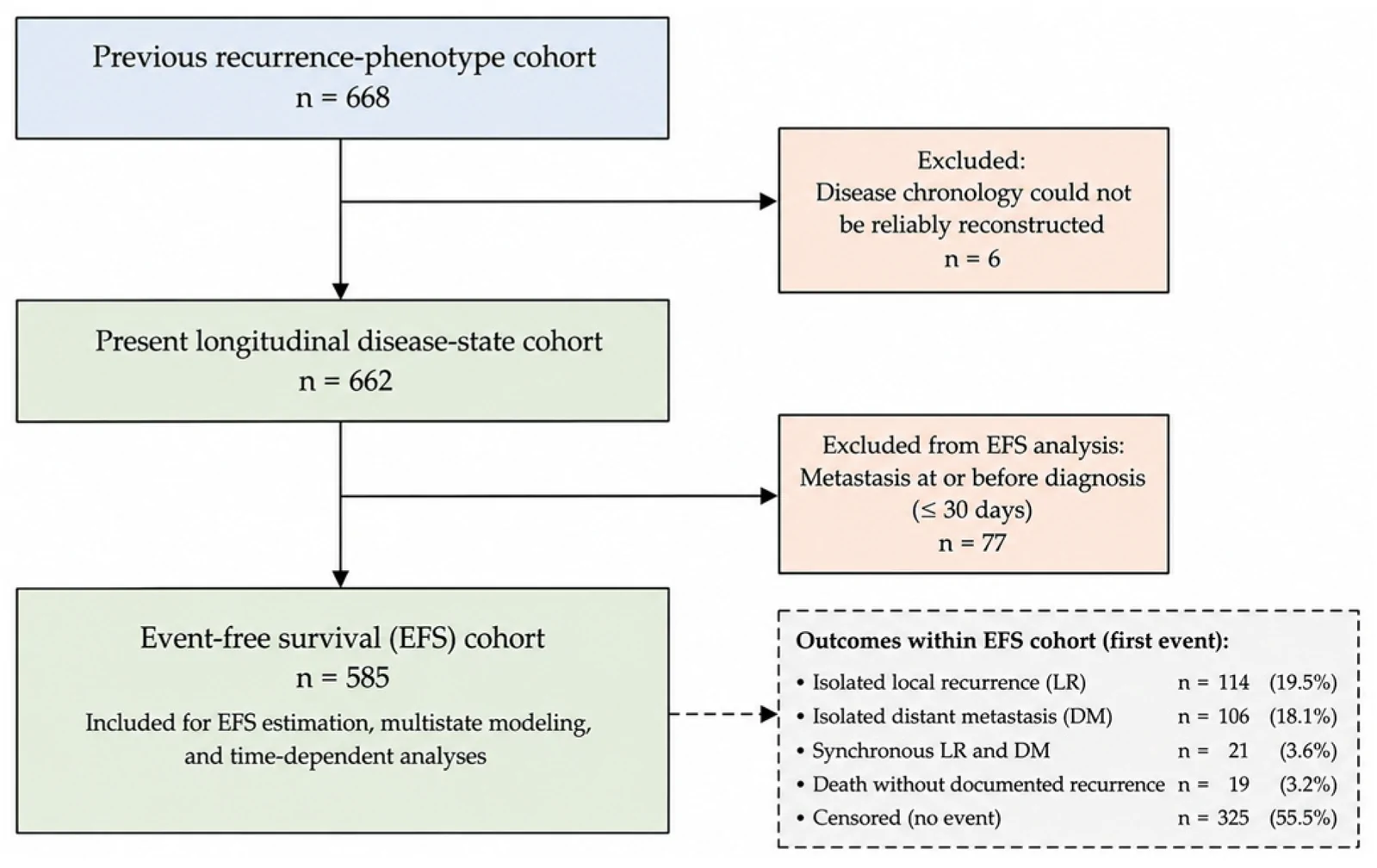
Flowchart of patient selection from the Swiss Sarcoma Network registry. Adapted from Ref. [25]; under the terms of the Creative Commons Attribution 4.0 International (CC BY 4.0) license.

Patients were excluded if they were treated outside the participating tertiary sarcoma centers, had benign tumors, primary bone sarcomas, or non-soft tissue sarcoma entities according to the World Health Organization (WHO) Classification of Soft Tissue and Bone Tumors [28,29]. Anatomical location was considered when distinguishing atypical lipomatous tumor (ALT) from well-differentiated liposarcoma (WDLS). ALT/WDLS was retained in the primary analysis because the study was designed to characterize the full spectrum of disease states encountered in tertiary sarcoma practice, including tumors associated predominantly with local rather than metastatic recurrence. Given the very low metastatic potential of peripheral ALT/WDLS, a sensitivity analysis excluding peripheral ALT/WDLS was performed to assess its influence on the principal findings. Histological subtypes were reported individually when considered clinically relevant. Less frequent entities were grouped as “Other.” Cases with incomplete longitudinal event information preventing reconstruction of the chronological sequence of LR, DM, and death were also excluded. The patient selection process is summarized in Figure 1.

The index date (time zero) was defined as the date of definitive primary surgery. For patients who did not undergo surgical treatment, the date of histological diagnosis was used as the index date. Patients without surgery were retained to preserve the real-world composition of the registry cohort, including patients managed non-operatively or presenting with metastatic disease. All patients were followed until death or the date of last documented clinical follow-up.

### 2.3. Definition of Dynamic Disease States

**Figure 2:**
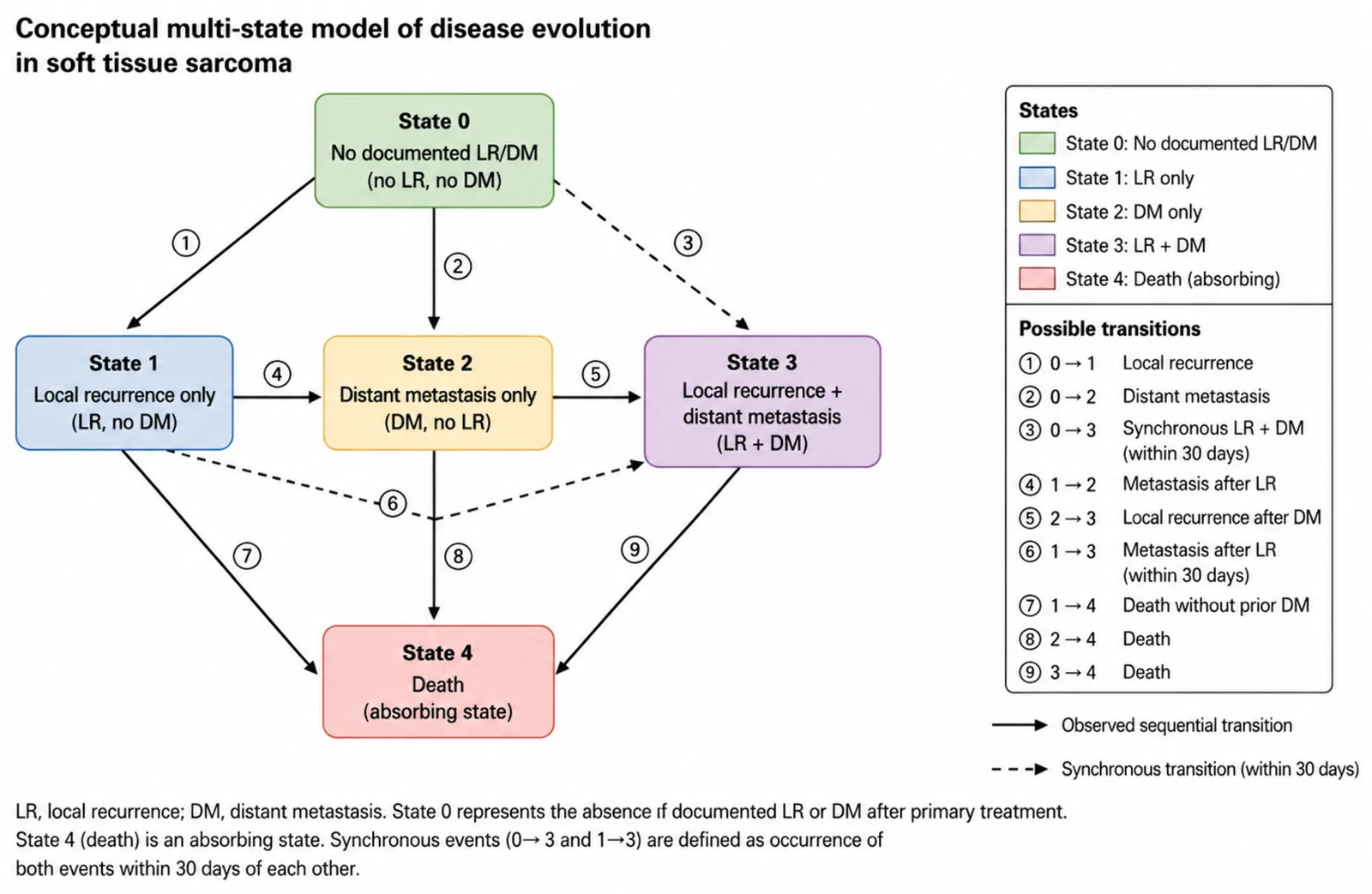
Conceptual multi-state model of disease progression in STS. Disease states and all permissible transitions used in the multi-state analysis are illustrated. Death represents the terminal absorbing state.

For the purposes of the multi-state model, State 0 was defined operationally as the absence of documented LR or DM after the index date and was termed “no documented LR or DM.” Patients who did not undergo surgery were retained to preserve the real-world composition of the registry cohort, including patients managed non-operatively and those presenting with metastatic disease. For these patients, the date of histological diagnosis was used as the index date. This operational state definition did not necessarily imply complete eradication of the primary tumor. LR was defined as histologically or radiologically confirmed tumor recurrence at or adjacent to the primary tumor site. DM was defined as metastatic disease at any distant anatomical site; nodal metastases and nodal recurrences were included in the metastatic disease state. Metastatic disease at presentation was defined as distant metastasis identified at diagnosis or within 30 days thereafter. Patients with metastatic disease at presentation entered the multi-state model directly in the metastatic state at time zero rather than transitioning from the no documented LR or DM state. Synchronous LR and DM were defined as the occurrence of both events within 30 days of each other; in these cases, the transition to the combined LR + DM state was assigned to the earlier of the two event dates. Patients were classified into one of eight mutually exclusive disease-state pathways according to the chronological sequence of LR and DM during follow-up (Figure 2).

### 2.4. Outcome Measures

The primary outcome was the longitudinal evolution of disease through predefined clinical disease states following definitive primary treatment. Disease progression was characterized by transitions between the no documented LR/DM, LR, DM, combined LR and DM, and death. Secondary outcomes included conventional EFS, transition-specific rates between disease states, and overall survival according to the patient’s current disease state. Death from any cause was included as an EFS event and represented the terminal absorbing state in the multi-state model. “Death without documented recurrence” was defined as death occurring before any documented LR or DM, irrespective of the recorded cause of death. Mortality was evaluated using time-dependent disease-state analyses to quantify the prognostic impact of evolving disease status over time.

### 2.5. Statistical Analysis

Baseline patient, tumor, and treatment characteristics were summarized using descriptive statistics. Continuous variables are presented as median and interquartile range (IQR), and categorical variables as frequencies and percentages. Group comparisons were performed using the Kruskal–Wallis test for continuous variables and the χ^2^ or Fisher’s exact test for categorical variables, as appropriate.

Conventional EFS was estimated using the Kaplan–Meier method, with events defined as the first occurrence of LR, DM, synchronous LR and DM, or death. Patients without an event were censored at the date of last follow-up.

To characterize longitudinal disease evolution, a progressive multi-state model comprising five disease states (no documented LR/DM, isolated LR, isolated DM, combined LR and DM, and death) was constructed. Patients contributed person-time sequentially to each occupied disease state until transition to the next state, censoring, or death. Transition-specific rates were calculated for all permissible transitions and are reported as events per 100 patient-years with corresponding 95% confidence intervals (CIs).

State-occupation probabilities were estimated nonparametrically using the Aalen–Johansen estimator, accounting for competing transitions between no documented LR/DM and treating death as an absorbing state. Probabilities of occupying each state were estimated over time and reported at prespecified landmark time points.

The association between evolving disease states and mortality was evaluated using Cox proportional hazards regression with disease state modeled as a time-dependent covariate. Hazard ratios (HRs) with 95% CIs were estimated relative to the no documented LR/DM state. An additional multivariable time-dependent Cox model was adjusted for age, tumor size, histological subtype, tumor grade, and surgical margin status. Missing covariate data were handled using complete-case analysis without imputation. These covariates were selected a priori based on established prognostic relevance in STS and their availability across the cohort. Patients who did not undergo surgery were retained as a separate category for surgical margin status rather than being classified as having missing margin data. The final multivariable model included 546 patients and 125 deaths. As a sensitivity analysis, the multivariable time-dependent Cox model was repeated after excluding patients with peripheral ALT/WDLS, using the same covariate set and complete-case approach.

The proportional hazards assumption was assessed using time-by-covariate interaction terms, and no material violation was identified. Model convergence and influential observations were also examined. All analyses were performed using SAS software version 9.4 (SAS Institute, Cary, NC, USA). All statistical tests were two-sided, and *p* < 0.05 was considered statistically significant.

## 3. Results

### 3.1. Study Cohort and Baseline Characteristics

Patients were identified from the SSN registry and underwent sequential screening according to the predefined eligibility criteria (Figure 1). Following exclusion of patients treated outside the participating tertiary sarcoma centers, benign tumors, primary bone sarcomas, non-soft tissue sarcoma entities according to the WHO Classification, and cases with incomplete event chronology, the final study cohort comprised 662 patients with histologically confirmed STS available for longitudinal analysis.

Median follow-up for the overall cohort was 50.0 months (interquartile range [IQR] 21.0–96.8 months), during which 151 deaths were recorded. Baseline demographic, tumor, and treatment characteristics are summarized in Appendix A Table A1.

The cohort reflected the biological heterogeneity typically observed in adult STS. Histological subtype, tumor grade, anatomical location, and tumor depth varied substantially across patients. Overall, deep-seated tumors predominated, and high-grade lesions represented a considerable proportion of the cohort. Liposarcoma, leiomyosarcoma, and undifferentiated pleomorphic sarcoma were among the most frequently represented histological subtypes.

As expected in a real-world multicenter cohort, treatment strategies also differed according to baseline tumor characteristics. Significant variation was observed in surgical margin status, use of radiotherapy, chemotherapy, and the frequency of unplanned (“whoops”) resections (Table A1B).

Overall, the study cohort encompassed a broad spectrum of clinicopathological presentations, providing the foundation for subsequent analyses examining how patients transition through distinct local and metastatic disease states over the course of follow-up.

### 3.2. Decomposition of the Conventional Event-Free Survival Endpoint Reveals Distinct First-Event Types

**Figure 3:**
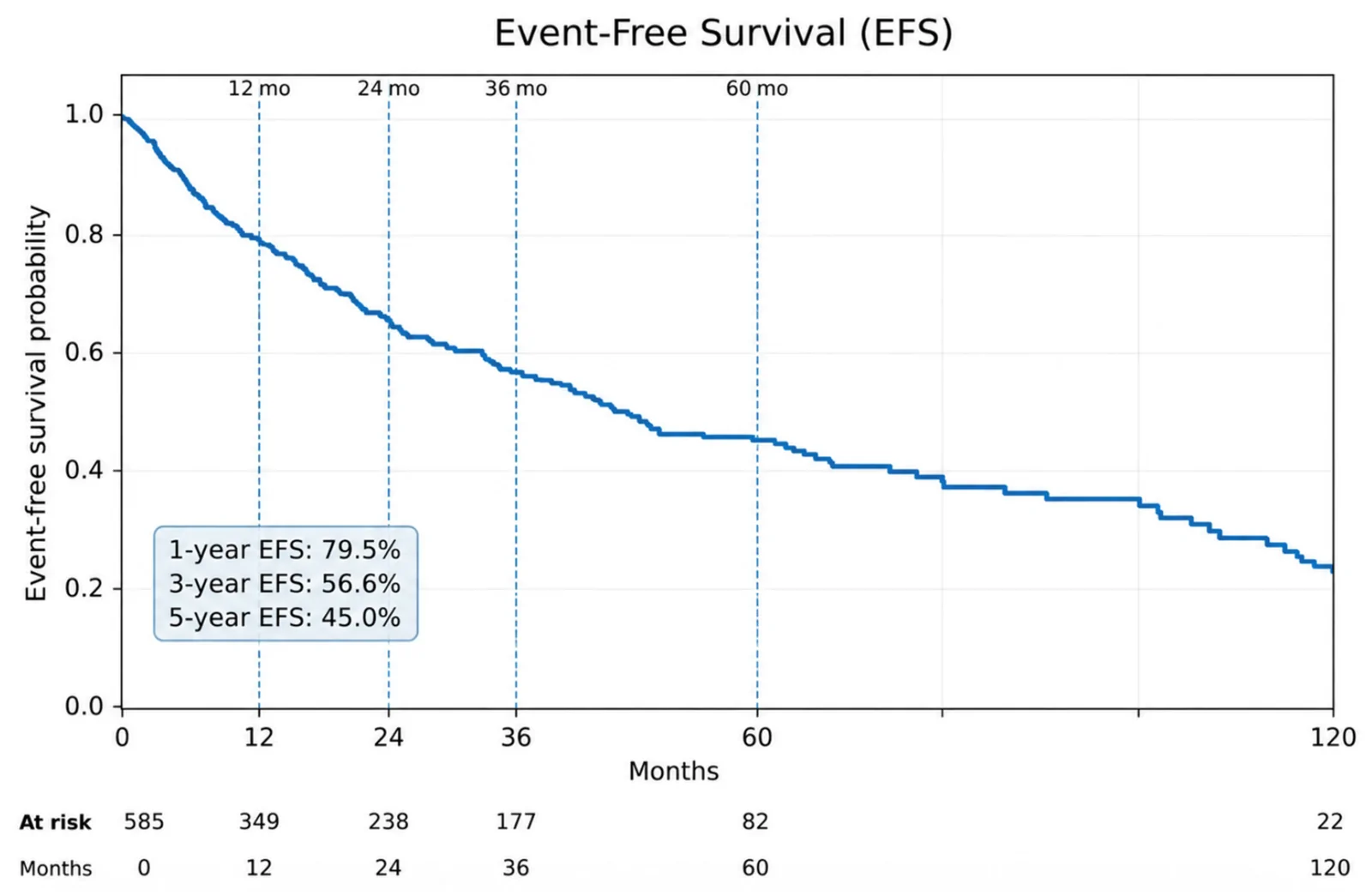
Kaplan–Meier event-free-survival curve for patients without metastatic disease at presentation (*n* = 585). EFS was defined as the time to first LR, DM, synchronous LR and DM, or death. One-, three-, and five-year EFS rates were 79.5%, 56.6%, and 45.0%, respectively. Numbers at risk are shown below the x-axis.

To examine the composition of the conventional EFS endpoint, analyses were restricted to the 585 patients who had no evidence of DM at presentation. During follow-up, estimated EFS was 79.5% at 1 year, 56.6% at 3 years, and 45.0% at 5 years (Figure 3).

Decomposition of the composite EFS endpoint demonstrated that first events represented several clinically distinct patterns of disease progression rather than a single homogeneous outcome (Table 1). LR was the initial event in 114 patients (19.5%), DM in 106 patients (18.1%), and synchronous LR and DM in 21 patients (3.6%). An additional 19 patients (3.2%) died without documented recurrence, whereas 325 patients (55.5%) remained free of LR and DM throughout follow-up.

Although LR and DM occurred with similar frequency as the first event, they reflected fundamentally different manifestations of disease progression. These findings demonstrate that the conventional EFS endpoint combines biologically distinct clinical events, providing the rationale for subsequent analyses evaluating disease evolution through dynamic transitions between local and metastatic disease states.

### 3.3. Observed Disease-State Pathways

**Figure A1:**
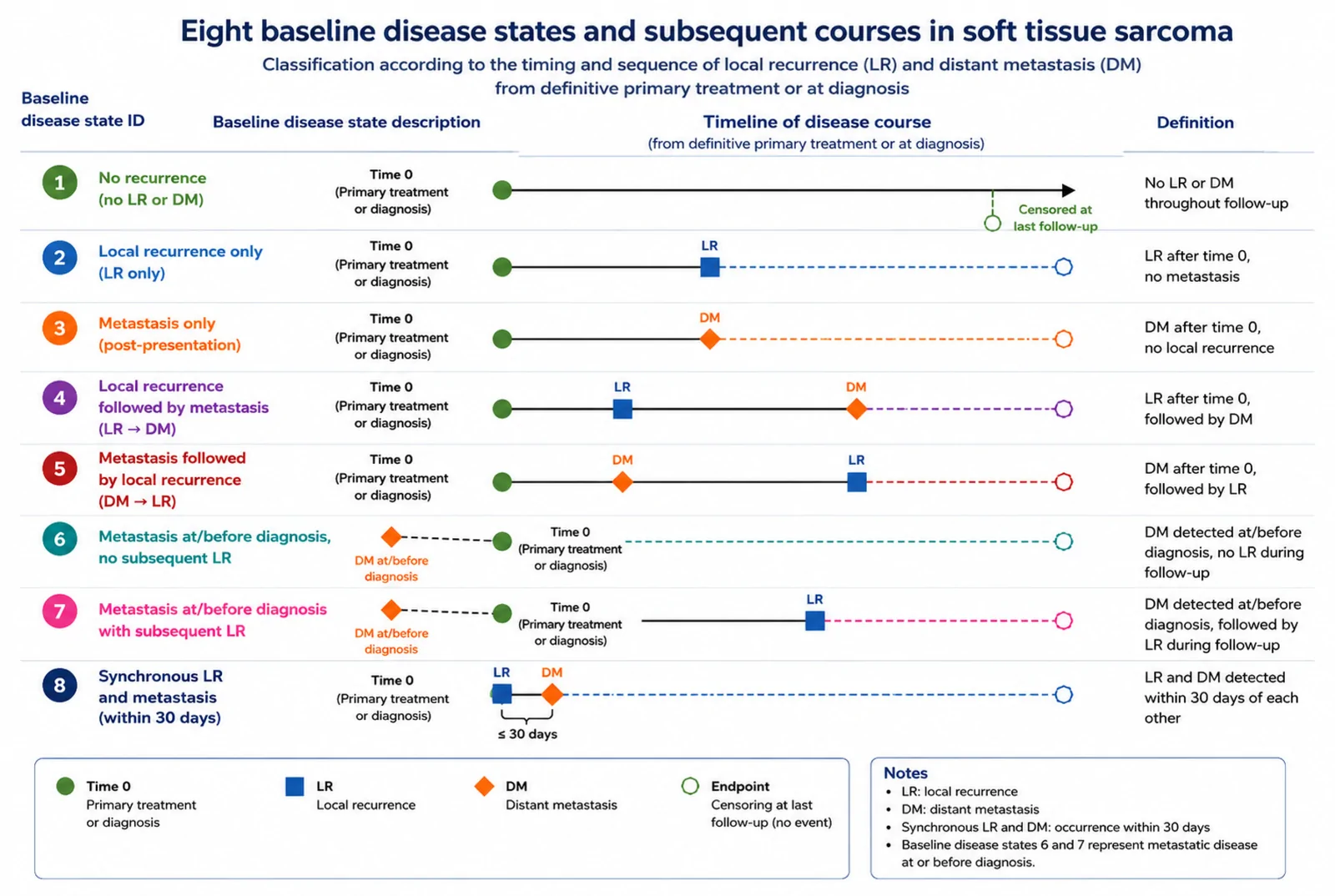
Observed disease-state pathways in soft tissue sarcoma. Eight mutually exclusive disease-state pathways describe the longitudinal evolution of local recurrence (LR) and distant metastasis (DM) from definitive primary treatment or diagnosis.

Following decomposition of the conventional EFS endpoint, eight distinct disease-state pathways describing the longitudinal evolution of LR and DM were identified (Appendix A Figure A1). Among the full cohort, 344 patients (52.0%) had no documented LR or DM during follow-up. This group comprised 325 patients who were censored without an EFS event and 19 patients who died before any documented LR or DM. Patients with metastatic disease at presentation were classified separately and were not included in this group.

Among patients experiencing disease progression after diagnosis, isolated LR (90, 13.6%) and isolated post-presentation DM (90, 13.6%) represented the most frequent pathways. Sequential progression from LR to DM occurred in 24 patients (3.6%), whereas 16 patients (2.4%) developed LR only after metastatic progression. At presentation, 68 patients (10.3%) had metastatic disease without subsequent LR, while 9 patients (1.4%) developed LR after presenting with metastases. Finally, 21 patients (3.2%) experienced synchronous LR and DM during follow-up.

Baseline clinicopathological characteristics differed significantly across disease-state pathways (Table A1A,B). The most pronounced differences were observed for tumor grade, histological subtype, tumor depth, and treatment modality (all *p* < 0.001), whereas age, sex, tumor size, and treating institution did not differ significantly between pathways. Patients remaining recurrence-free were enriched for grade 1 liposarcoma, while metastatic pathways were dominated by grade 3 tumors, deep tumor location, and aggressive histological subtypes, particularly leiomyosarcoma and undifferentiated pleomorphic sarcoma. Consistent with these clinicopathological differences, radiotherapy and chemotherapy were used more frequently in patients who subsequently developed metastatic disease (both *p* < 0.001). These observed disease-state pathways formed the descriptive basis for the subsequent multistate analyses.

### 3.4. Dynamic Transitions Between Disease States

To characterize disease evolution beyond the first event, patients were modelled as transitioning between predefined disease states representing no documented LR/DM, isolated LR, isolated DM, combined LR and DM, and death. Transition-specific rates are summarized in Table 2.

Among patients initially with no documented LR/DM, transitions to isolated LR and isolated DM occurred at comparable rates, with transition rates of 7.46 (95% CI, 6.21–8.96) and 6.93 (95% CI, 5.73–8.39) events per 100 patient-years, respectively. Direct progression from the no documented LR/DM state to synchronous LR and DM (1.37 per 100 patient-years; 95% CI, 0.90–2.11) or death (1.24 per 100 patient-years; 95% CI, 0.79–1.95) was uncommon.

Following isolated LR, progression to combined LR and DM occurred at a transition rate of 4.77 (95% CI, 3.20–7.12) events per 100 patient-years, whereas the corresponding rate for death remained low (1.79 per 100 patient-years; 95% CI, 0.93–3.44). In contrast, patients with isolated DM transitioned either to combined LR and DM (7.59 per 100 patient-years; 95% CI, 5.13–11.23) or directly to death (25.50 per 100 patient-years; 95% CI, 20.59–31.58) at substantially higher rates. Likewise, patients with combined LR and DM demonstrated a similarly high rate of death (26.01 per 100 patient-years; 95% CI, 19.00–35.60).

Overall, transition rates remained low while disease was confined to isolated LR but increased markedly following metastatic progression, highlighting the dynamic prognostic shift associated with DM.

### 3.5. State-Occupation Probabilities over Time

**Figure 4:**
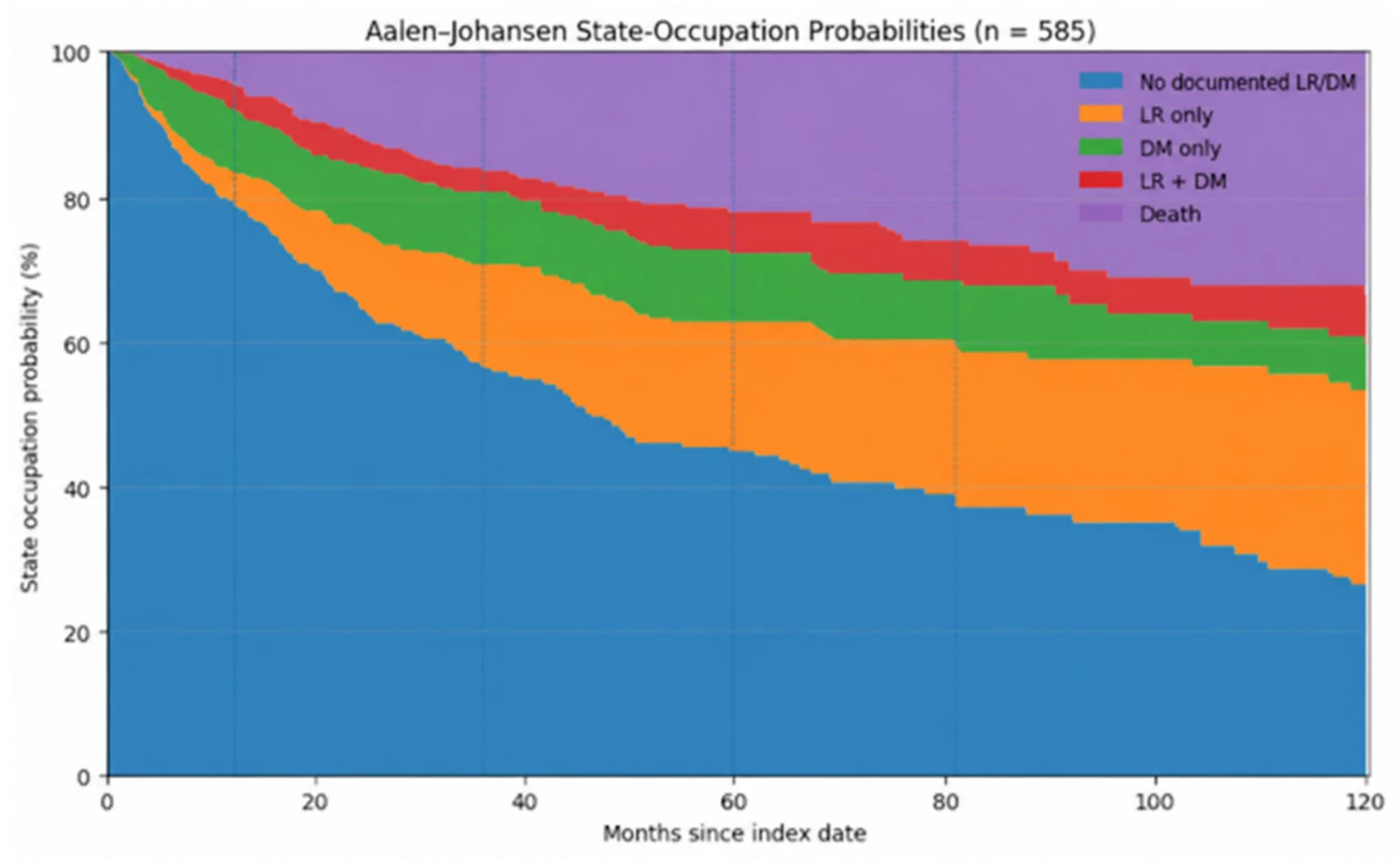
Aalen–Johansen state-occupation probabilities over time among patients entering follow-up in the no documented LR/DM state (*n* = 585).

Aalen–Johansen estimates demonstrated a progressive redistribution of patients across disease states during follow-up (Figure 4). Among the 585 patients entering the analysis in the no documented LR/DM state, the probability of remaining disease-free decreased from 79.5% at 12 months to 56.6% at 36 months and 45.0% at 60 months. At 60 months, the estimated probabilities of occupying the disease-free, LR-only, DM-only, combined LR and DM, and death states were 44.9%, 17.9%, 9.2%, 5.9%, and 22.1%, respectively. These findings illustrate the progressive diversification of disease states over time beyond the first-event information captured by conventional EFS.

### 3.6. Current Disease States and Mortality

To evaluate how prognosis changed according to patients’ evolving disease status, mortality risk was assessed using the patient’s current disease state as a time-dependent exposure, with the disease-free state serving as the reference category (Table 3).

In the unadjusted analysis, isolated LR was associated with a moderate increase in mortality risk (HR 3.21, 95% CI 1.36–7.61; *p* = 0.008). In contrast, mortality increased markedly following metastatic progression. Patients with isolated DM had a 23-fold higher hazard of death (HR 23.29, 95% CI 14.14–38.37; *p* < 0.001), while patients with combined LR and DM experienced the highest mortality risk (HR 40.09, 95% CI 22.61–71.08; *p* < 0.001).

After adjustment for age at diagnosis, tumor size, histological subtype, tumor grade, and surgical margin status, the prognostic gradient across disease states persisted. Isolated LR showed a moderate but statistically imprecise association with mortality (HR 2.55, 95% CI 0.93–7.02; *p* = 0.070). In contrast, the adjusted mortality hazard remained markedly higher for isolated DM (HR 17.56, 95% CI 9.46–32.60; *p* < 0.001) and combined LR and DM (HR 32.63, 95% CI 16.86–63.17; *p* < 0.001).

After exclusion of 92 patients with peripheral ALT/WDLS, the adjusted current-state model included 467 patients and 123 deaths. The prognostic gradient was preserved, with an HR of 2.79 for isolated LR (95% CI 1.00–7.79; *p* = 0.051), 17.92 for isolated DM (95% CI 9.48–33.87; *p* < 0.001), and 33.40 for combined LR and DM (95% CI 16.88–66.08; *p* < 0.001).

These findings demonstrate a pronounced prognostic gradient across evolving disease states, with metastatic progression—particularly combined local and metastatic disease—associated with the greatest mortality risk.

### 3.7. Time-Dependent Cox Regression Analysis

To account for dynamic changes in disease status over time and minimize immortal time bias, a time-dependent Cox regression model was constructed in which LR and DM were included as separate time-dependent covariates. Both recurrence events were independently associated with mortality. The occurrence of LR was associated with an approximately two-fold increase in the hazard of death (HR 1.88, 95% CI 1.29–2.74; *p* = 0.001), whereas the development of metastatic disease was associated with a nearly 20-fold increase in mortality risk (HR 19.86, 95% CI 13.04–30.24; *p* < 0.001). These findings corroborate the disease-state analysis, demonstrating that although both LR and DM were associated with mortality, metastatic progression showed by far the strongest association with mortality.

## 4. Discussion

### 4.1. Principal Findings

In this multicenter cohort of 662 patients with STS, disease progression was more accurately represented as a sequence of dynamic disease states than as a single EFS endpoint. Decomposition of conventional EFS in the 585-patient disease-free survival cohort revealed substantial heterogeneity among first events: isolated LR occurred first in 114 patients (19.5%), DM in 106 (18.1%), synchronous LR and DM in 21 (3.6%), and death without documented recurrence in 19 (3.2%). Although these events contribute equally to conventional EFS, they were followed by markedly different disease courses and survival outcomes, consistent with previous evidence that local and metastatic recurrence represent clinically distinct manifestations of STS progression [17,25,30,31,32].

Longitudinal analyses further demonstrated that prognosis evolved according to the patient’s current disease state. In time-dependent Cox analysis, the prognostic gradient across disease states persisted after adjustment for age at diagnosis, tumor size, histological subtype, tumor grade, and surgical margin status.

Isolated LR showed a moderate but statistically imprecise association with mortality after adjustment (HR 2.55, 95% CI 0.93–7.02), whereas substantially greater mortality hazards were observed for isolated DM (HR 17.56, 95% CI 9.46–32.60) and combined LR and DM (HR 32.63, 95% CI 16.86–63.17). These findings emphasize that recurrence events are not prognostically equivalent, with metastatic progression showing by far the strongest association with mortality. Together, these findings support a disease-state-based framework that complements conventional EFS by capturing the changing prognostic significance of STS progression over time [3,22,23].

### 4.2. Beyond Conventional Event-Free Survival: Heterogeneity Within Composite Endpoints

Conventional EFS is widely used in STS research because combining multiple clinically relevant outcomes into a single composite endpoint improves statistical efficiency [7,8]. However, this approach treats biologically and prognostically distinct events as equivalent. Composite endpoints may therefore obscure clinically meaningful differences between event types, particularly when their mechanisms, therapeutic consequences, and associations with subsequent survival differ substantially [11]. Moreover, the individual components of composite endpoints are often insufficiently reported, further limiting their clinical interpretation [7].

Our findings illustrate this limitation in STS. Isolated LR and DM occurred as first events with comparable frequencies (19.5% and 18.1%, respectively), yet their subsequent clinical implications differed markedly. Isolated LR was associated with comparatively favorable outcomes, whereas metastatic progression was accompanied by a profound increase in mortality. This distinction is consistent with previous studies identifying LR and DM as clinically distinct manifestations of disease progression and is reflected in current management, where isolated LR may remain amenable to curative treatment, whereas metastatic disease generally requires systemic disease control [17,32,33]. Thus, events contributing equally to EFS may represent fundamentally different clinical states [34].

Conventional EFS should therefore not necessarily be replaced, but complemented by approaches that preserve the nature and longitudinal evolution of its component events. Multi-state and semi-competing risk models retain information lost when heterogeneous outcomes are collapsed into a single endpoint. By decomposing EFS and modeling subsequent disease-state transitions, recurrence can be interpreted as part of an evolving longitudinal process rather than simply as the first occurrence of an event, potentially improving prognostic interpretation and the design and analysis of future STS studies [3,11,35].

### 4.3. Dynamic Disease States Reveal the Longitudinal Evolution of Soft Tissue Sarcoma

Our findings demonstrate that STS progression is best understood as a continuum of evolving disease states rather than as a sequence of isolated recurrence events. This was reflected in the marked differences in mortality across disease states. These findings indicate that the prognostic significance of recurrence depends not only on its occurrence, but also on the disease state subsequently occupied [18,19].

The multi-state framework captures this longitudinal process by preserving transitions between clinically distinct states and their changing prognostic implications over time. Previous multistate analyses in STS have similarly demonstrated progressively worsening outcomes across transitions from local to metastatic disease and ultimately death [4,36,37]. Our findings extend these observations by showing that prognosis is continuously updated as patients move between disease states, with metastatic progression representing the major shift toward adverse outcome.

This disease-state perspective may also provide a basis for more dynamic prognostic assessment. Recent prediction models, including PERSARC and BayeSarc, illustrate the potential value of incorporating newly acquired clinical information to update individualized survival estimates during follow-up [22,36]. Rather than treating recurrence as the endpoint of disease-free survival, each disease state can therefore be considered an intermediate stage within an evolving disease process, providing a framework for longitudinal risk assessment and potentially more individualized clinical decision-making.

### 4.4. Clinical Implications of a Dynamic Disease-State Framework

The marked prognostic differences between disease states have potential implications for surveillance, prognostic counseling, and therapeutic decision-making in STS. In the adjusted time-dependent analysis, isolated LR showed a moderate but statistically imprecise association with mortality, whereas metastatic disease—either alone or in combination with LR—remained strongly associated with mortality. However, the magnitude of this association differed markedly according to disease state. Isolated LR was associated with a moderate increase in mortality, whereas metastatic disease—either alone or in combination with LR—was associated with substantially greater mortality risk. These findings suggest that risk assessment should be reconsidered as patients transition between disease states rather than relying exclusively on clinicopathological characteristics established at diagnosis or on the occurrence of recurrence itself. This principle is consistent with current clinical practice guidelines, which distinguish between isolated LR, where curative treatment remains feasible in selected patients, and metastatic disease, where management is primarily directed toward systemic disease control [34,38].

A disease-state framework may therefore complement conventional surveillance and prognostic assessment by incorporating information acquired during follow-up. Rather than assigning patients to a fixed risk category at diagnosis, their prognostic profile can be updated as disease evolves. Such an approach could support more individualized patient counseling and provide a conceptual basis for adapting surveillance and treatment strategies to the patient’s current disease status. However, prospective validation is required before disease-state-based surveillance strategies can be recommended for routine clinical practice [3,10,39,40].

The framework also has implications for clinical research. Explicitly modeling transitions between clinically distinct states provides information that conventional time-to-first-event analyses cannot capture and may improve patient stratification, endpoint interpretation, and longitudinal prognostic assessment in observational studies and clinical trials [22,23,36,37]. Dynamic prediction models such as PERSARC and BayeSarc illustrate how updated clinical information can be incorporated into individualized risk estimates over time [41]. Integrating disease-state transitions into future prognostic models may therefore provide a more clinically representative approach to risk prediction in STS [42,43,44].

### 4.5. Strengths and Limitations

A principal strength of this study is the integration of EFS decomposition, longitudinal disease-state classification, multi-state modeling, and time-dependent survival analyses within a unified analytical framework. The study was based on a multicenter sarcoma registry combining prospectively recorded clinical information with retrospectively abstracted medical-record data, enabling reconstruction of disease evolution beyond the first recurrence event. The relatively large cohort of 662 patients further allowed characterization of multiple clinically relevant disease states in this rare and heterogeneous malignancy [45,46]. Unlike our previous phenotype-based analysis, the present study provides a cohort-level description of longitudinal disease-state transitions and state-dependent mortality and was not developed or validated as an individual-patient prediction model.

Several limitations should be considered. First, because the study included retrospectively abstracted data and was analyzed retrospectively, it remains susceptible to residual confounding and selection bias, particularly within tertiary referral centers [16,22]. Unmeasured factors, including comorbidity, performance status, disease burden, and treatment selection, may also have influenced mortality and were not fully captured by the adjusted models. Given the observational study design, the hazard ratios should be interpreted as measures of association and not as causal effects of entering a particular disease state. Referral patterns, retrospective reconstruction of some clinical events, and differences in management between centers may therefore limit the generalizability of the findings beyond specialized sarcoma settings. Second, some disease-state subgroups, particularly patients with synchronous LR and DM or LR following metastatic disease, were small, limiting the precision of subgroup-specific estimates. These pathway-specific findings should therefore be interpreted descriptively and with appropriate caution. Third, external validation in independent cohorts is required to establish the generalizability and transportability of the proposed framework [47,48]. Accordingly, the framework should not be considered ready for broader clinical implementation until it has been evaluated in independent populations.

Finally, death represents a competing event for subsequent LR, particularly in patients with aggressive metastatic disease, potentially leading to underestimation of later LR in rapidly progressive disease [39,49,50]. The observed distribution of disease states and transitions should therefore be interpreted within the context of competing risks.

Despite these limitations, the marked prognostic differences across disease states and the concordant findings of the multi-state and time-dependent analyses support the internal consistency of the observed disease-state framework [47,48,49,50].

## 5. Conclusions

This study demonstrates that STS progression is more accurately represented as a sequence of dynamic disease states than as a single event-free survival endpoint. By decomposing conventional EFS and modeling longitudinal transitions between LR, metastatic disease, and death, we identified distinct disease states with markedly different prognostic implications. This framework complements conventional time-to-first-event analyses by capturing the evolving nature of disease progression and may improve longitudinal prognostic assessment and interpretation of clinical outcomes. Future studies should validate this approach in independent cohorts and evaluate its potential integration into clinical decision-making. Together, these findings support a transition from event-based to disease-state-based assessment of soft tissue sarcoma progression.

## Figures and Tables

**Table 1:** First-event decomposition of event-free survival in patients without metastatic disease at presentation (*n* = 585). EFS events comprised LR, DM, synchronous LR and DM, or death. Synchronous events were defined as LR and DM occurring within 30 days.

First EFS Event	*n*	%
LR first	114	19.5%
Metastasis first	106	18.1%
Synchronous LR + metastasis	21	3.6%
Death without recurrence	19	3.2%
Censored/no event	325	55.5%

**Table 2:** Transition-specific rates within the multi-state model. Rates are expressed as events per 100 patient-years (95% CI). State 0 indicates no documented LR/DM, state 1 isolated local recurrence (LR), state 2 isolated distant metastasis (DM), state 3 combined LR and DM, and state 4 death.

*Transition*	*Clinical Interpretation*	*Events (n)*	*Rate/100 PY (95% CI)*
*0* *→* *1*	No documentet LR/DM → local recurrence	114	7.46 (6.21–8.96)
*0* *→* *2*	No documentet LR/DM → metastasis	106	6.93 (5.73–8.39)
*0* *→* *3*	No documentet LR/DM → synchronous LR + metastasis	21	1.37 (0.90–2.11)
*0* *→* *4*	No documentet LR/DM → death	19	1.24 (0.79–1.95)
*1* *→* *3*	LR only → LR + metastasis	24	4.77 (3.20–7.12)
*1* *→* *4*	LR only → death	9	1.79 (0.93–3.44)
*2* *→* *3*	Metastasis only → LR + metastasis	25	7.59 (5.13–11.23)
*2* *→* *4*	Metastasis only → death	84	25.50 (20.59–31.58)
*3* *→* *4*	LR + metastasis → death	39	26.01 (19.00–35.60)

**Table 3:** Time-dependent Cox regression analysis evaluating mortality according to the patient’s current disease state. The no documented LR/DM state served as the reference category. The adjusted model included age at diagnosis, tumor size, histological subtype, tumor grade, and surgical margin status and was based on complete cases (*n* = 546; 125 deaths). HR, hazard ratio; CI, confidence interval; LR, local recurrence; DM, distant metastasis.

Current Disease State	Unadjusted HR (95% CI)	*p*	Adjusted HR (95% CI)	*p*
No documented LR/DM	Reference	—	Reference	—
LR only	3.21 (1.36–7.61)	0.008	2.55 (0.93–7.02)	0.070
Metastasis only	23.29 (14.14–38.37)	<0.001	17.56 (9.46–32.60)	<0.001
LR + metastasis	40.09 (22.61–71.08)	<0.001	32.63 (16.86–63.17)	<0.001

## Data Availability

The data presented in this study are available from the corresponding author upon reasonable request, subject to applicable ethical and data-protection restrictions.

## Appendix A

**Table A1:** (**A**) Baseline clinicopathological characteristics according to observed disease-state pathway. Continuous variables are presented as median (interquartile range [IQR]) and categorical variables as *n* (%). Disease-state pathways were defined by the temporal sequence of local recurrence (LR) and distant metastasis (DM). Other histological subtypes include all remaining sarcoma subtypes with low individual frequencies. (**B**) Treatment characteristics according to observed disease-state pathway. Values are presented as *n* (%). Treatment allocation reflects routine multidisciplinary sarcoma care and should be interpreted descriptively rather than as a causal determinant of subsequent disease progression.

**(A)**									
***Variable***	***No Event*** ***(n = 344)***	***LR Only*** ***(n = 90)***	***Met Only*** ***(n = 90)***	***LR* *->* *Met*** ***(n = 24)***	***Met* *-> LR*** ***(n = 16)***	***Met at Pres.*** ***(n = 68)***	***Met at Pres. + LR*** ***(n = 9)***	***Sync LR + Met*** ***(n = 21)***	***p-Value***
***Institution***									0.686
*Institution 1*	196 (57.0%)	49 (54.4%)	57 (63.3%)	14 (58.3%)	11 (68.8%)	39 (57.4%)	3 (33.3%)	13 (61.9%)	
*Institution 2*	148 (43.0%)	41 (45.6%)	33 (36.7%)	10 (41.7%)	5 (31.2%)	29 (42.6%)	6 (66.7%)	8 (38.1%)	
*Age at diagnosis*	61.0 (49.0–73.0)	61.5 (48.2–72.8)	58.0 (46.0–72.8)	60.5 (47.8–67.5)	58.5 (51.8–62.0)	63.5 (50.2–76.5)	66.0 (56.0–71.0)	59.0 (50.0–72.0)	0.596
***Sex***									0.111
*male*	188 (54.7%)	50 (55.6%)	40 (44.4%)	10 (41.7%)	7 (43.8%)	30 (44.1%)	8 (88.9%)	10 (47.6%)	
*female*	156 (45.3%)	40 (44.4%)	50 (55.6%)	14 (58.3%)	9 (56.2%)	38 (55.9%)	1 (11.1%)	11 (52.4%)	
***Histology***									<0.001
*Angiosarcoma*	13 (3.8%)	0 (0.0%)	4 (4.4%)	4 (16.7%)	2 (12.5%)	9 (13.2%)	0 (0.0%)	0 (0.0%)	
*Leiomyosarcoma*	33 (9.6%)	4 (4.4%)	28 (31.1%)	1 (4.2%)	4 (25.0%)	17 (25.0%)	1 (11.1%)	4 (19.0%)	
*Liposarcoma*	158 (46.0%)	47 (52.2%)	19 (21.1%)	8 (33.4%)	3 (18.8%)	12 (17.7%)	0 (0.0%)	6 (28.6%)	
*MPNST*	2 (0.6%)	2 (2.2%)	0 (0.0%)	0 (0.0%)	1 (6.2%)	2 (2.9%)	0 (0.0%)	1 (4.8%)	
*Myxofibrosarcoma*	22 (6.4%)	10 (11.1%)	2 (2.2%)	2 (8.3%)	1 (6.2%)	1 (1.5%)	0 (0.0%)	0 (0.0%)	
*Other*	74 (21.6%)	17 (18.9%)	12 (13.3%)	3 (12.5%)	1 (6.2%)	12 (17.6%)	3 (33.3%)	2 (9.6%)	
*Rhabdomyosarcoma*	6 (1.8%)	0 (0.0%)	0 (0.0%)	1 (4.2%)	0 (0.0%)	4 (5.9%)	2 (22.2%)	1 (4.8%)	
*Synovial sarcoma*	7 (2.0%)	1 (1.1%)	4 (4.4%)	1 (4.2%)	0 (0.0%)	1 (1.5%)	1 (11.1%)	1 (4.8%)	
*Undifferentiated/pleomorphic sarcoma*	29 (8.4%)	9 (10.0%)	21 (23.3%)	4 (16.7%)	4 (25.0%)	10 (14.7%)	2 (22.2%)	6 (28.6%)	
***Grade***									<0.001
*G1*	167 (48.5%)	34 (37.8%)	5 (5.6%)	4 (16.7%)	1 (6.2%)	5 (7.4%)	0 (0.0%)	4 (19.0%)	
*G2*	78 (22.7%)	21 (23.3%)	19 (21.1%)	4 (16.7%)	3 (18.8%)	12 (17.6%)	2 (22.2%)	5 (23.8%)	
*G3*	99 (28.8%)	35 (38.9%)	65 (72.2%)	16 (66.7%)	12 (75.0%)	51 (75.0%)	7 (77.8%)	11 (52.4%)	
*n/a*	0 (0.0%)	0 (0.0%)	1 (1.1%)	0 (0.0%)	0 (0.0%)	0 (0.0%)	0 (0.0%)	1 (4.8%)	
***Tumor size***	75.0 (43.2–137.5)	80.0 (45.0–180.0)	82.0 (54.8–124.2)	80.0 (57.0–156.0)	105.0 (61.2–130.0)	105.0 (54.0–141.5)	130.0 (66.0–200.0)	105.0 (80.0–160.0)	0.199
***Region***									<0.001
*axial*	48 (14.0%)	13 (14.4%)	9 (10.0%)	4 (16.7%)	3 (18.8%)	4 (5.9%)	0 (0.0%)	1 (4.8%)	
*face, head and neck*	16 (4.7%)	2 (2.2%)	6 (6.7%)	4 (16.7%)	0 (0.0%)	5 (7.4%)	0 (0.0%)	0 (0.0%)	
*intra- and retroperitoneal*	53 (15.4%)	23 (25.6%)	21 (23.3%)	6 (25.0%)	5 (31.2%)	23 (33.8%)	5 (55.6%)	8 (38.1%)	
*lower extremity*	151 (43.9%)	32 (35.6%)	31 (34.4%)	5 (20.8%)	3 (18.8%)	25 (36.8%)	3 (33.3%)	5 (23.8%)	
*upper extremity*	52 (15.1%)	15 (16.7%)	11 (12.2%)	5 (20.8%)	2 (12.5%)	4 (5.9%)	0 (0.0%)	2 (9.5%)	
*urogenital, perianal, and anal*	24 (7.0%)	5 (5.6%)	12 (13.3%)	0 (0.0%)	3 (18.8%)	7 (10.3%)	1 (11.1%)	5 (23.8%)	
***Depth***									<0.001
*deep*	265 (77.0%)	76 (84.4%)	82 (91.1%)	19 (79.2%)	15 (93.8%)	64 (94.1%)	9 (100.0%)	21 (100.0%)	
*superficial*	79 (23.0%)	14 (15.6%)	8 (8.9%)	5 (20.8%)	1 (6.2%)	4 (5.9%)	0 (0.0%)	0 (0.0%)	
**(B)**									
***Variable***	***No Event*** ***(n = 344)***	***LR Only*** ***(n = 90)***	***Met Only*** ***(n = 90)***	***LR -> Met*** ***(n = 24)***	***Met -> LR*** ***(n = 16)***	***Met at Pres.*** ***(n = 68)***	***Met at Pres. + LR*** ***(n = 9)***	***Sync LR + Met*** ***(n = 21)***	***p-Value***
***Whoops surgery***									0.029
*no*	249 (72.4%)	55 (61.1%)	73 (81.1%)	15 (62.5%)	15 (93.8%)	54 (79.4%)	5 (55.6%)	14 (66.7%)	
*yes*	94 (27.3%)	33 (36.7%)	15 (16.7%)	8 (33.3%)	1 (6.2%)	14 (20.6%)	4 (44.4%)	7 (33.3%)	
*n/a*	1 (0.3%)	2 (2.2%)	2 (2.2%)	1 (4.2%)	0 (0.0%)	0 (0.0%)	0 (0.0%)	0 (0.0%)	
***Margin status***									<0.001
*R0*	240 (69.8%)	28 (31.1%)	62 (68.9%)	7 (29.2%)	6 (37.5%)	18 (26.5%)	3 (33.3%)	12 (57.1%)	
*R1*	57 (16.6%)	33 (36.7%)	9 (10.0%)	6 (25.0%)	8 (50.0%)	8 (11.8%)	4 (44.4%)	6 (28.6%)	
*R2*	4 (1.2%)	7 (7.8%)	4 (4.4%)	5 (20.8%)	0 (0.0%)	6 (8.8%)	1 (11.1%)	2 (9.5%)	
***No Surgery***	35 (10.2%)	0 (0.0%)	6 (6.7%)	0 (0.0%)	0 (0.0%)	30 (44.1%)	0 (0.0%)	0 (0.0%)	
*n/a*	8 (2.3%)	22 (24.4%)	9 (10.0%)	6 (25.0%)	2 (12.5%)	6 (8.8%)	1 (11.1%)	1 (4.8%)	
***Radiotherapy***									<0.001
*no*	207 (60.2%)	48 (53.3%)	19 (21.1%)	5 (20.8%)	2 (12.5%)	33 (48.5%)	4 (44.4%)	5 (23.8%)	
*yes*	137 (39.8%)	42 (46.7%)	71 (78.9%)	19 (79.2%)	14 (87.5%)	35 (51.5%)	5 (55.6%)	16 (76.2%)	
***Chemotherapy***									<0.001
*no*	323 (93.9%)	79 (87.8%)	41 (45.6%)	10 (41.7%)	3 (18.8%)	30 (44.1%)	2 (22.2%)	7 (33.3%)	
*yes*	21 (6.1%)	11 (12.2%)	49 (54.4%)	14 (58.3%)	13 (81.2%)	38 (55.9%)	7 (77.8%)	14 (66.7%)	
